# Identification of potential metabolic biomarkers of polycystic ovary syndrome in follicular fluid by SWATH mass spectrometry

**DOI:** 10.1186/s12958-019-0490-y

**Published:** 2019-06-11

**Authors:** Zhengao Sun, Hsun-Ming Chang, Aijuan Wang, Jingyan Song, Xingxing Zhang, Jiayin Guo, Peter C. K. Leung, Fang Lian

**Affiliations:** 1 Integrative Medicine Research Centre of Reproduction and Heredity, The Affiliated Hospital of Shandong University of Traditional Chinese Medicine, No 42 Wen Hua Xi Road, Jinan, 250011 China; 2 Department of Obstetrics and Gynaecology, BC Children’s Hospital Research Institute, University of British Columbia, Vancouver, British Columbia V6H 3V5 Canada; 3 Guandong Provincial Key Laboratory of New Drug Screening, School of Pharmaceutical Sciences, Southern Medical University, Guangzhou, 510515 China; 4 Department of Obstetrics and Gynaecology, BC Children’s Hospital Research Institute, University of British Columbia, Room 317, 950 West 28th Avenue, Vancouver, British Columbia V5Z 4H4 Canada

**Keywords:** Follicular fluid, IVF-ET, Metabolomics, SWATH, PCOS

## Abstract

**Background:**

Polycystic ovary syndrome (PCOS) is a complex disorder associated with multiple metabolic disturbance, including defective glucose metabolism and insulin resistance. The altered metabolites caused by the related metabolic disturbance may affect ovarian follicles, which can be reflected in follicular fluid composition. The aim of this study is to investigate follicular fluid metabolic profiles in women with PCOS using an advanced sequential window acquisition of all theoretical fragment-ion spectra (SWATH) mass spectrometry.

**Materials and methods:**

Nineteen women with PCOS and twenty-one healthy controls undergoing IVF/ET were recruited, and their follicular fluid samples were collected for metabolomic study. Follicular fluid metabolic profiles, including steroid hormones, free fatty acids, bioactive lipids, and amino acids were analyzed using the principal component analysis (PCA) and partial least squares to latent structure-discriminant analysis (PLS-DA) model.

**Results:**

Levels of free fatty acids, 3-hydroxynonanoyl carnitine and eicosapentaenoic acid were significantly increased (*P* < 0.05), whereas those of bioactive lipids, lysophosphatidylcholines (LysoPC) (16:0), phytosphingosine, LysoPC (14:0) and LysoPC (18:0) were significantly decreased in women with PCOS (*P* < 0.05). Additionally, levels of steroid hormone deoxycorticosterone and two amino acids, phenylalanine and leucine were higher in the PCOS patients (*P* < 0.05).

**Conclusion:**

Women with PCOS display unique metabolic profiles in their follicular fluid, and this data may provide us with important biochemical information and metabolic signatures that enable a better understanding of the pathogenesis of PCOS.

## Introduction

Polycystic ovary syndrome (PCOS) is a common endocrine disorder that occurs among women of reproductive age [1]. Recent studies have identified multiple factors that are involved in the pathogenesis of PCOS, including endocrine factors and genetic factors [2]. Women with PCOS have a high risk of female subfertility, type II diabetes, cardiovascular diseases, and endometrial cancers [3]. Despite extensive studies, there is still no unified, objective, and modern biological standard for diagnosing PCOS. Therefore, previous clinical studies have produced few effective treatment schemes that are based on pathogenesis and dialectics. Given the lack of in-depth knowledge regarding this disorder, it is necessary to provide new breakthrough research methods to circumvent these shortcomings.

Metabolomics study is an effective approach to identify metabolic alterations and quantify endogenous cellular metabolites in organisms [4]. This postgenomic approach has been applied to rapidly identify global metabolic changes in biological systems and is a useful tool to discover biomarkers, diagnose diseases, identify perturbed pathways, and measure responses to medical treatment [5]. Gaining a better understanding of the pathogenesis of PCOS and enabling the identification of potential biomarkers will significantly advance our ability to prevent, obtain early and accurate diagnosis of, and effectively treat women with PCOS. Previous studies performed using ultra-performance liquid chromatography quadrupole time-of-flight mass spectrometry (UPLC-Q-TOF) have demonstrated that several metabolites are differentially expressed in serum and urine in PCOS [6–8]. The results showed that serum free fatty acids, dihydrotestosterone sulfate, and urine glycated phenylalanine could serve as potential biomarkers for diagnosing PCOS. However, whether there are characteristic differences in metabolite levels in follicular fluid between PCOS patients and healthy volunteers has not been explored. Follicular fluid is derived from ovarian follicles, which contain many types of metabolites that are involved in many critical processes of follicular development and oocyte maturation. Studies have shown that alteration in the homeostasis of the microenvironment of the follicular fluid is related to polycystic ovaries [9], PCOS [10], and reproductive aging [11, 12].

Although studies performed using high-resolution mass spectrometry have become a powerful strategy for metabolomics analyses, low-level differential endogenous metabolites are still very difficult to investigate [13, 14]. The hit rates of the fragment ion spectrum play an important role in identifying differentially expressed endogenous metabolites. Compared to the traditional information dependent acquisition (IDA) method [15, 16] and the mass spectrometry (MS^ALL^) technique, sequential window acquisition of all theoretical fragment-ion spectra (SWATH™) can significantly improve the hit rate of low-level endogenous metabolites [17]. This new technique can sequentially obtain all MS/MS spectra of full mass windows across the specified mass range and has relatively higher specificity and sensitivity, as was previously reported in a study of proteomics and metabolomics [18]. Another advantage of the SWATH™ acquisition technique is that researchers can reprocess the same data set to identify other previously unidentified features without reacquiring the samples [19].

In this study, we used a nontarget follicular fluid metabolomics method based on the SWATH technique to identify many differential metabolites in women with PCOS; this is the first study to use this approach. We aimed to increase understanding of the complex metabolic process that occur in patients with PCOS.

## Materials and methods

### Experimental chemicals

The internal standards, including isotope-labeled 18:1(d7) monoglyceride (MAG), isotope-labeled 15:0–18:1(d7) diglyceride (DAG), isotope-labeled 18:1(d9) sphingomyelin (SM), isotope-labeled 18:1(d7) lysophosphatidylethanolamine (LPE), isotope-labeled d3-palmitic acid and Isotope-labeled 18:1(d7) lysophosphatidylcholine (LPC), were purchased from Sigma-Aldrich (St. Louis, MO, USA). Distilled water was obtained from a Milli-Q system (Millipore, MA, USA). Chromatographic grade formic acid, acetonitrile and methanol were purchased from Fisher (Fairlawn, NJ, USA).

### Sample collection and preparation

Patients with PCOS (*n* = 19) and healthy women (*n* = 21, as a control group) were recruited from the Reproductive and Genetic Center of Integrated Traditional and Western Medicine at Shandong University of Traditional Chinese Medicine Affiliated Hospital, from January to December 2016. The study was approved by the Health Authorities and Ethics Committees of Shandong University of Traditional Chinese Medicine Affiliated Hospital. All subjects signed an informed consent form prior to the study. Patients with PCOS were diagnosed based on the Rotterdam 2003 criteria, which includes the exclusion of other endocrine disorders and requires two of the following three features to be met: 1) oligo- or anovulation, 2) clinical and/or biochemical signs of hyperandrogenism, or 3) polycystic ovaries [20]. The control group consisted of subjects with infertility purely due to a male factor (azoospermia or severe oligo−/asthenoteratozoospermia). Women with infertility due to poor ovarian reserve, ovulatory dysfunction, tubal factor, and endometriosis were excluded from the control group.

All subjects underwent controlled ovarian hyperstimulation according to our established protocols. Once more than three follicles were larger than 18 mm, 10,000 IU human chorionic gonadotropin was intramuscularly administered, and the mature follicles (diameter ≥ 18 mm) were aspirated using 17-gauge Cook needles. Oocytes were subsequently retrieved. After the oocytes were separated, follicular fluid obtained from 3 dominant follicles was pooled and centrifuged at 10,000×g for 30 min to remove insoluble particles and cells. The supernatant was then stored at − 80 °C until further study.

### Serum hormone measurement and follicle calculation

Circulating levels of hormones, including serum FSH, LH, E2, testosterone (detected at day 2), E2 and P (detected at hCG day), were measured using a radioimmunoassay method. The numbers of antral follicles were counted using ultrasonography on day 2.

### Measurement of the reproducibility and accuracy of SWATH mass spectrometry

Before analysis, eleven follicular fluid samples were thawed at room temperature for quality control (QC) (one QC after every four follicular fluid samples). First, samples were prepared by mixing 100 μL of each individual follicular fluid samples. To reduce the effect of the solvent and obtain a good peak shape, a total volume of 150 μL of follicular fluid or QC sample was mixed with 450 μL of methanol (v/v, 1:3) containing isotope-labeled 18:1(d7)MAG, isotope-labeled 15:0–18:1(d7)DAG, isotope-labeled d3-palmitic acid, isotope-labeled 18:1(d9)SM, isotope-labeled 18:1(d7)LPE and isotope-labeled 18:1(d7)LPC. Next, the mixture was vortexed for 10 min and centrifuged at 13000×g for 20 min at 15 °C. The contents of the supernatant were analyzed using UPLC-Q-TOF.

### Method conditions

Aliquots of 5 μL of the supernatant were injected into the UPLC tandem Triple TOF 5600 system (SCIEX, CA, USA) in random order. A reverse-phase 2.1*100 mm ACQUITY 1.7 μm C_18_ column (Waters, Ireland) was used for separation. A gradient mobile phase composed of 0.05% formic acid solution (A) and acetonitrile (B) was used and kept at 90% A for 0.5 min, increased to 95% B over the next 6.5 min, and then returned to 90% A from 8.5 min to 8.6 min. The total running time was 13 min. The mass parameters were as follows: nebulizing gas, 55 psi; TIS gas, 55 psi; source temperature, 500 °C; and ion spray voltage, 5000 V with 35 psi curtain gas in positive mode and − 4000 V with 35 psi curtain gas in negative mode. The declustering potential and collision energy were set at 55 V and 40 ± 20 V, respectively, in positive mode (− 55 V and − 40 ± 20 V, respectively, in negative mode). The SWATH method with 20 variable isolation windows was performed in TOF MS full-scan mode and in TOF MS/MS product ion scan mode at m/z 50–1200 in Analyst TF 1.7.1 software.

### Data collection, processing and statistical analysis

SPSS 22.0 statistical software was used. The continuous data with a normal distribution are statistically described as the means ± standard and were compared by independent sample t-tests. The classification data are statistically described using frequency (composition ratio) and chi-square tests. *P* < 0.05 was considered statistically significant for the two groups of data tested.

Data were acquired by the workstation of the instrument and peak extraction, and alignment was checked using MarkerView software (SCIEX, CA, USA). The full width at half maximum (FWHM) was set to 12, and the retention time window was set to 10. Other parameters were default values. The normalization preprocessing method was used to decrease systematic changes for our large-scale nontargeted LC-MS metabolomics measurements. The normalization effect was assessed using the internal standards including isotope-labeled 18:1(d7)MAG, isotope-labeled 15:0–18:1(d7)DAG, isotope-labeled 18:1(d9)SM, isotope-labeled 18:1(d7)LPE, isotope-labeled 18:1(d7)LPC and isotope-labeled d3-palmitic acid. Detected and matched peaks with m/z values, retention times and their corresponding intensities were exported to a text table. The zero-values were removed with the modified 80% rule. A multivariate statistical analysis was performed to reflect the differences between patients with PCOS and healthy individuals using SIMCA-P software version 11.0 (Umetrics AB, Umea, Sweden). Principal component analysis (PCA) and partial least squares-discriminant analysis (PLS-DA) models were constructed with the reversed-phase liquid chromatography data. The number of significant components was calculated using cross-validation (seven times). Chemical noise was avoided using the Pareto scaling in all the models. In a loading plot, each point represented an ion that contributed to the sample separation between patients with PCOS and healthy individuals. The differential metabolic ions that exerted a major influence on the group membership were selected according to the variable importance in the project (VIP) value and the loading plot. These metabolites were considered potential biomarkers and identified according to accurate mass, isotope patterns, and fragmentation patterns.

## Results

### The demographic and clinical characteristics of women with PCOS and non-PCOS controls

The average age, BMI, total number of antral follicles, serum E2 and P levels on day 2 and the number of retrieved oocytes of the participants are shown in Table 1. There was no significant difference in the average age and BMI between the two groups. Compared with non-PCOS patients, the average total number of antral follicles, serum E2 and P levels on day 2 and the number of retrieved oocytes were significantly higher in patients with PCOS.

**Table 1:** The demographic and clinical characteristics of women with PCOS and non-PCOS control

Item	PCOS group (*n* = 19)	Control group (*n* = 21)	*p* value
Age (y)	30.1 ± 2.3	32.5 ± 4.1	0.24
BMI (kg/m^2^)	23.9 ± 4.4	22.7 ± 2.3	0.29
Duration of infertility (y)	1.6 ± 1.5	1.5 ± 1.4	0.83
Primary infertility, n (%)	8 (42.1%)	10 (47.6%)	0.73
FSH (mIU/ml)*	6.6 ± 1.6	7.4 ± 1.0	0.31
LH (mIU/ml)*	5.3 ± 3.5	6.0 ± 8.7	0.75
E_2_ (pg/ml)*	42.7 ± 25.2	35.9 ± 15.0	0.30
T (ng/ml)*	0.50 ± 0.49	0.36 ± 0.23	0.25
AFC*	20.5 ± 10.1	10.2 ± 5.7	< 0.001
E2 on HCG day(pg/ml)	4375.9 ± 1203.8	2420.6 ± 1411.9	< 0.001
P on HCG day(ng/ml)	1.6 ± 0.6	1.2 ± 0.5	0.03
Days of gonadotropin use (d)	11.3 ± 2.8	10.6 ± 3.4	0.48
Total Gonadotropin dose (U)	2109.3 ± 815.5	2941.9 ± 1173.6	0.01

### Reproducibility and accuracy of the SWATH-MS system

**Fig. 1:**
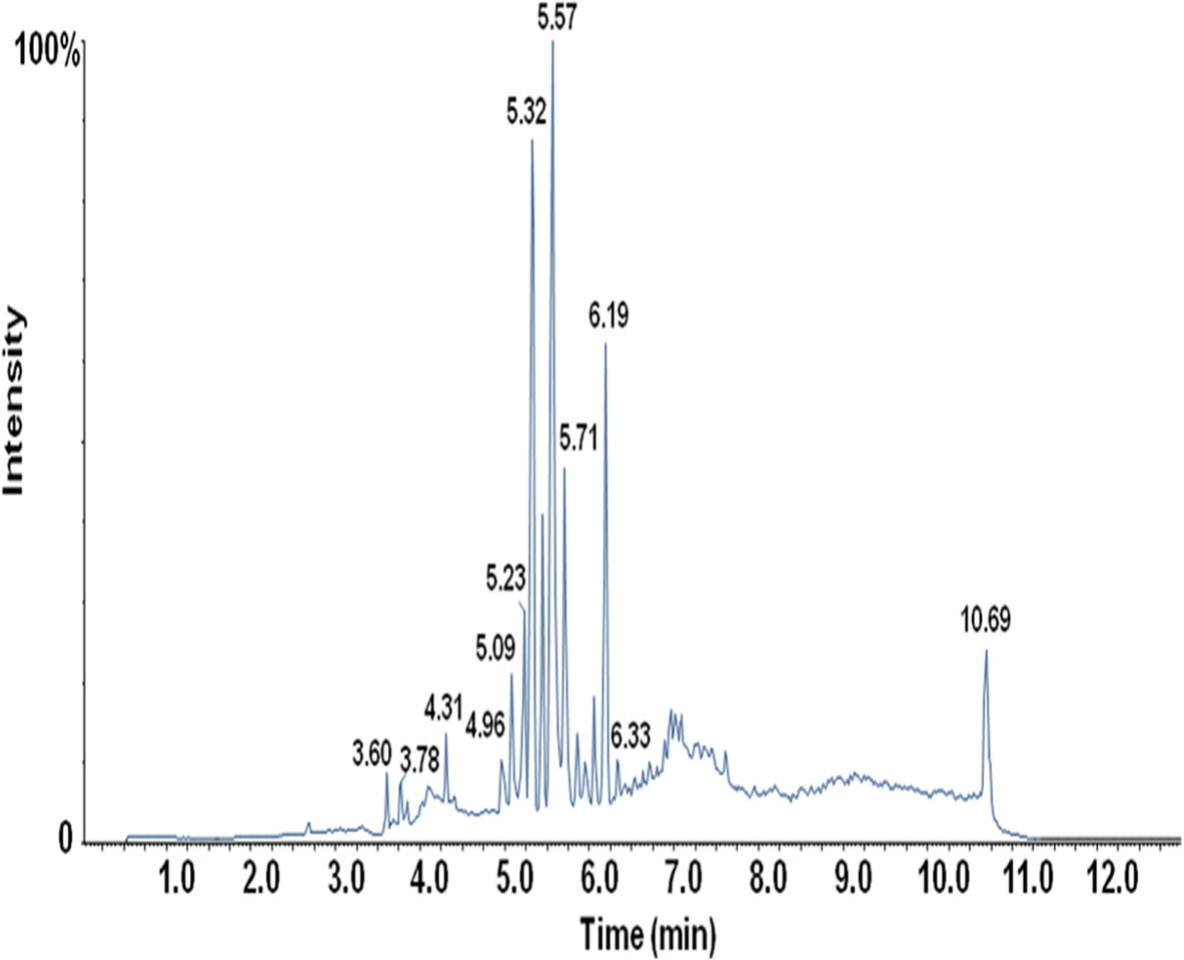
Total ion chromatogram of one representative QC follicular fluid sample. The x-axis and y-axis represent for the retention time and total ion intensity, respectively

A previous study by Ron Bonner *et. al*. summarized that SWATH is well suited to QqTOF instruments for metabolomics study compared to the conventional approach such as Data-Dependent Acquisition (DDA) [21]. There were 5408 compound features using SWATH technique and 3942 compound features using DDA technique in follicular fluid in our study. More compound features were detected using SWATH technique than DDA technique. Before the analyses of all of the follicular fluid samples, pooled quality control (QC) sample obtained from 11 normal follicular fluid samples was prepared by mixing an equal volume of follicular fluid samples and six internal standards. QC samples were used to assess the reproducibility and reliability of the SWATH-MS system. One QC sample was run at every 4 samples. The total ion chromatogram of one follicular fluid QC sample was shown in Fig. 1. The chromatographic separation was good. The peaks of six internal standards were chosen to evaluate the reproducibility of the analysis method. The relative standard deviation (RSD) of the peak areas within the batch was less than 11.3%, indicating that the reproducibility and reliability of this detection system were good according to the FDA guidelines. The m/z of internal standard ions was detected to be less than 1 ppm compared to the theoretical mass, indicating that the accuracy of this method is adequate to detect unknown samples. Next, we assessed the chromatography residue using the peak area of the internal standard in the blank sample following the follicular fluid sample. The internal standard peaks were not found in blank sample, indicating that there was no chromatography residue. Collectively, these data suggest that this analytical method is reliable and accurate for metabolomics studies.

**Fig. 2:**
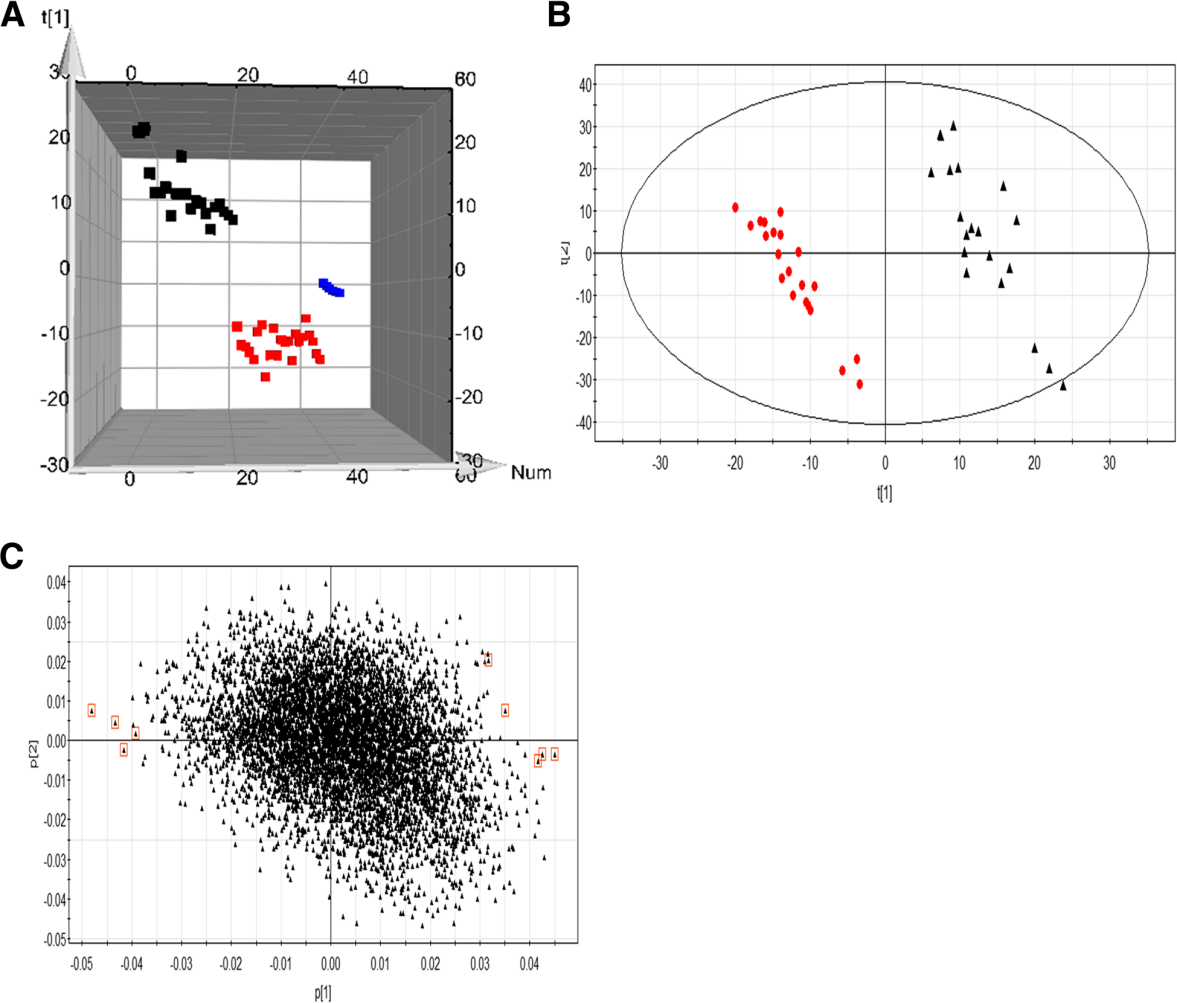
**a** PCA 3D-score plots based on UPLC-Q-TOF data. PCOS group (black spots), Control group (red spots), and QC group (blue spots). **b** The score plot shows the PLS-DA model. PCOS group (black spots) and Control group (red spots). **c** The loading plot shows the PLS-DA model. Each point represents for one metabolite ion. The points marked with red boxes are the potential differential metabolites ions

Before multivariate statistical analysis was performed, peaks with a retention time less than 0.6 min were excluded. PCA was used as an unsupervised statistical method to study the metabolomic differences among different groups. The 3D-score plots are shown in Fig. 2a. The PCOS group was completely separated from the healthy non-PCOS group. QC samples were tightly clustered together.

### Multivariate data analysis and selection of potential metabolite biomarkers in the follicular fluid of women with PCOS

To investigate the metabolic differences between the PCOS group and the non-PCOS group and to further identify potential biomarkers, we applied the supervised PLS-DA model using PCA for the overview of the metabolomic data set and the spotting of outliers. As shown in Fig. 2b, the results of the PLS-DA model performed using the data from UPLC-Q-TOF showed that the score plots of PCA analysis for all metabolites in follicular fluid samples were clearly separated between the PCOS and control groups. The parameters (predictive ability) used to describe the PLS-DA models were significantly elevated (R2Y = 0.79, Q2Y = 0.62), suggesting that the PLS-DA models were robust [16, 22]. These findings suggest that the levels of some metabolites were significantly changed in the follicular fluid of women with PCOS.

The VIP value reflects the influence of each metabolite ion on the classification. Variables with a VIP value > 3 (calculated using SIMCA-P) have an above-average influence on the explanation of the Y matrix. The metabolic ions with a VIP value > 3 are marked with a red square. Finally, 9 potential metabolic biomarkers were found and are shown in a loading plot (Fig. 2c). These potential metabolites exhibited significant changes (*P* < 0.05) based on the T-test statistical analysis.

### Identification of potential metabolites responsible for discrimination between women with and without PCOS

**Fig. 3:**
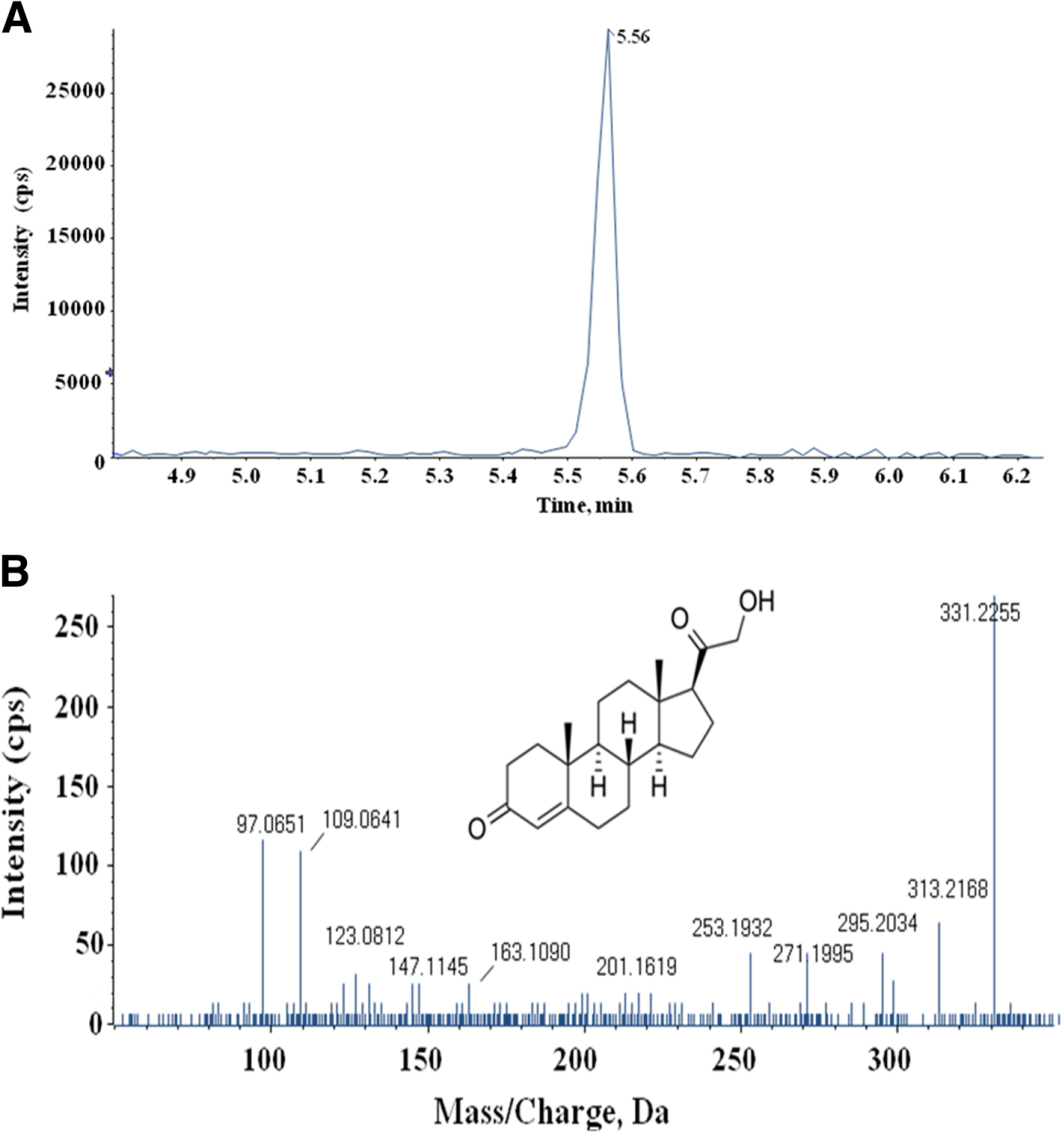
**a** The chromatograph of M1 (deoxycorticosterone). The x-axis and y-axis represent for the retention time and ion intensity, respectively. **b** The fragment ion spectrum of M1 (deoxycorticosterone). The x-axis and y-axis represent for the mass-to-charge ratio and ion intensity, respectively

**Fig. 4:**
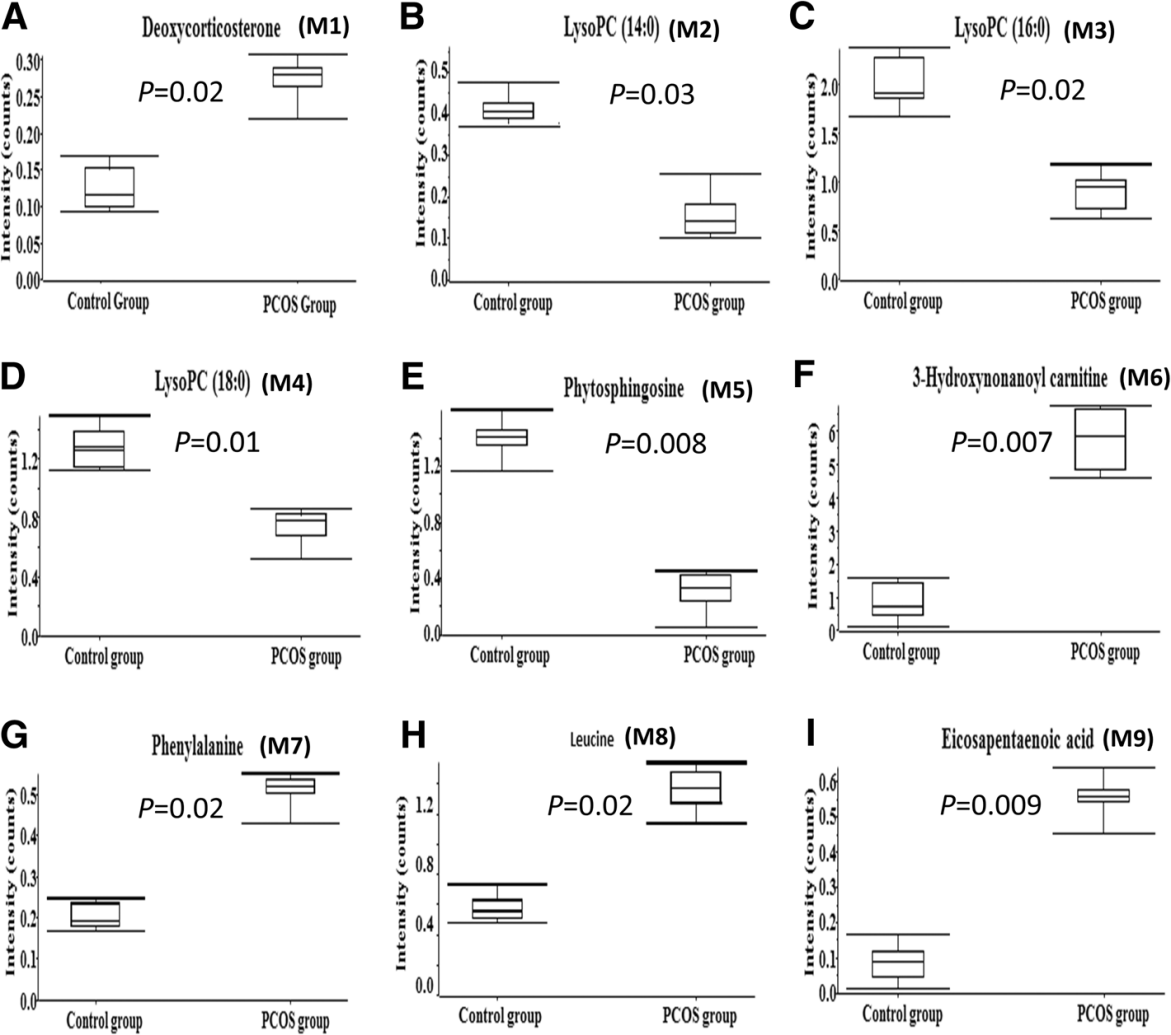
**a-i** Differential metabolite profiles of the nine candidate biomarkers identified in the quantitative analysis of the subjects (control and PCOS groups). The y-axis represents for the content of candidates. The box plot consists of the median (horizontal line) and the inter-quartile range, and the whiskers indicate the minimum and maximum values unless there are outliers, in which case the whiskers extend to a maximum of 1.5 times the inter-quartile range

A total of 9 potential metabolic biomarkers were identified using SWATH mass spectrometry. These metabolites were validated based on accurate mass, isotope patterns, and mass spectrometric fragmentation patterns. For example, the differential metabolite M1 was the [M + H]^+^ ion at *m/z* 331.2247. The elution time of M1 was 5.56 min in UPLC chromatography (Fig. 3a). Its molecular formula was inferred as C_21_H_30_O_3_ based on its accurate mass and isotope patterns. A series of characteristic productions at m/z 313.2114, 271.2037, 177.1229, 123.0785, 109.0641, and 97.0657 by a successive loss of H_2_O, C_2_H_4_O_2_, C_9_H_14_O_2_, C_13_H_20_O_2_, C_14_H_22_O_2_ and C_15_H_22_O_2_ were observed (Fig. 3b). Fragmentation patterns were also used to search METLIN and HMDB databases. The structure of M1 was inferred as deoxycorticosterone.. The detail results are shown in Table 2. These differences in the 9 metabolites between the two groups are displayed with Graph Pad Prism. Among the differential metabolites in follicular fluid, the levels of deoxycorticosterone, 3-hydroxynonanoyl carnitine, phenylalanine, eicosapentaenoic acid, and leucine were significantly higher, whereas the levels of lysoPC (14:0), lysoPC (16:0), lysoPC (18:0), and phytosphingosine were significantly lower in the PCOS group than in the non-PCOS control group (Fig. 4).

**Table 2:** Characteristics of the metabolic biomarkers of women with PCOS in follicular fluid using UPLC-Q-TOF MS

Metabolites	T_R_ (min)	Error (ppm)	Parent ion (m/z)	Molecular Formula	Product ion (m/z)	Identity (PCOS group vs Control group)	t-test (*p* value)
M1	5.56	−2.6	331.2247	C_21_H_30_O_3_	313.2114; 271.2037; 177.1229; 123.0785; 109.0641; 97.0657	Deoxycorticosterone	0.02
M2	4.84	−3.6	468.3068	C_22_H_46_NO_7_P	450.2971; 285.2384; 184.0727; 124.9999; 104.1078; 86.0955	LysoPC (14:0)	0.03
M3	5.72	3.7	496.3416	C_24_H_50_NO_7_P	313.2724; 184.0743; 125.0000; 104.1088; 86.0981	LysoPC (16:0)	0.02
M4	6.51	−1.7	524.3702	C_26_H_54_NO_7_P	506.3571; 341.3052; 184.0738; 125.0003; 104.1085; 86.0996	LysoPC (18:0)	0.01
M5	4.77	−2.4	318.2995	C_18_H_39_NO_3_	300.2859; 256.2627; 88.0777	Phytosphingosine	0.008
M6	6.85	−0.9	318.2272	C_16_H_31_NO_5_	300.2186; 274.2398; 160.0965	3-Hydroxynonanoyl carnitine	0.007
M7	2.88	3.3	166.0868	C_9_H_11_NO_2_	120.0808; 103.0544; 91.0563; 77.0394	Phenylalanine	0.02
M8	2.54	3.7	132.1024	C6H13NO2	86.0982; 69.0721	Leucine	0.02
M9	8.38	−3.0	301.2164	C_20_H_30_O_2_	257.1133; 59.0115	Eicosapentaenoic acid	0.009

*T*_*R*_ retention time

## Discussion

In the present study, we differentiated the characteristics of metabolic ions in follicular fluid in women with PCOS and healthy women and identified several potential metabolic biomarkers for this chronic disease.

Previous studies have shown that free fatty acids are critical molecular indicators because they are products of abnormal lipid metabolism that exert pronounced effects on cell growth, differentiation and metabolism by modulating gene expression [23, 24]. In mammals, the composition of fatty acids in the oocyte and the concentration of fatty acids in the surrounding environment may influence the developmental competence of oocytes and subsequent embryo implantation [25, 26]. These abnormal metabolites have been proposed to induce multiple stress markers of the endoplasmic reticulum that are detrimental to mammalian oocytes [27]. Women with PCOS have several systematic metabolic disturbances, including defective glucose metabolism and insulin resistance, which may affect ovarian follicles, and these effects may be reflected in follicular fluid composition. Indeed, studies performed using follicular fluid analysis in women with PCOS have demonstrated that defective glucose transportation and insulin resistance can induce a series of alternative energy pathways that produce altered follicular fluid concentrations of various bioproducts, including lipids, amino acids, and ketone bodies [28, 29]. Additionally, several spectroscopy-based studies have reported that specific free fatty acid concentrations in the serum and follicular fluid are altered in women with PCOS [27, 30]. Specifically, the concentrations of palmitoleic acid and oleic acid were higher in both the serum and follicular fluid in obese PCOS than in control and nonobese PCOS patients [27]. Additionally, serum concentrations of linoleic acid (C18:2n6cis) were higher in obese PCOS patients [27]. Furthermore, the concentrations of oleic acid and stearic acid were associated with oocyte developmental competence, which may account for the decreased pregnancy rate in women with PCOS [27]. Because fatty acids are a class of metabolites that could be used to evaluate stearoyl-CoA desaturase activity, the increased unsaturated fatty acids observed in PCOS indicate that stearoyl-CoA desaturase activity could be a potential marker for this disease [31]. In line with these findings, the data obtained from our studies showed that the follicular fluid concentrations of 3-hydroxynonanoyl carnitine and eicosapentaenoic acid were significantly increased in women with PCOS.

Previous studies have shown that women with PCOS have increased levels of the total endogenous amino acids that are associated with the clinical features of polycystic ovary and anovulation, suggesting an elevated degradation rate of proteins during ovarian dysfunction [32, 33]. In particular, PCOS patients with ovulatory dysfunction have elevated levels of the following five amino acids: phenylalanine, serine, threonine, tyrosine, and ornithine [32]. Another phenotype of women with PCOS is marked impairment of the tricarboxylic acid (TCA) cycle [32], which is characterized by a drop in succinyl-CoA and fumarate and increases in serum levels of phenylalanine, valine, threonine, and tyrosine [32]. Our studies also revealed that two amino acids, phenylalanine and leucine, were higher in the PCOS group (Fig. 4). Consistent with our results, two studies showed that leucine levels were higher in PCOS patients than in normal controls [34, 35].

In addition to metabolic abnormalities, PCOS patients experience a complex and heterogeneous endocrine disorder. Approximately one-third of PCOS patients have elevated serum androgen levels or clinical manifestations of hyperandrogenism [36]. In the current study, we found that follicular fluid concentrations of deoxycorticosterone were higher in women with PCOS, indicating that steroid hormone biosynthesis could be influenced in this disorder. The increase in follicular fluid deoxycorticosterone could be due to the metabolites of adrenal steroids, which are modulated by selective adrenal factors. The overresponse of adrenal androgen to adrenocorticotrophic hormone stimulation and increased hydroxylation activity could be the causes of the excess adrenal androgen observed in affected patients. However, the regulatory mechanism underlying this effect remains unclear. Future studies performed using animal models to elucidate the detailed molecular mechanisms underlying these effects will be required.

A recent study used gas chromatography–mass spectrometry and liquid chromatography mass spectrometry to analyze serum lipid profiles of women with PCOS [37]. That study showed that serum levels of polyunsaturated fatty acids (such as arachidonic acid, linoleic acid, and docosahexaenoic acid) and lysophospholipids were lower in obese patients with PCOS than in lean controls [37]. These results indicate that two common characteristics, obesity and hyperinsulinemia, observed in women with PCOS may stimulate the production of bioactive lipids. Similarly, our data showed that women with PCOS have lower follicular fluid levels of lysophosphatidylcholines (LysoPC) (16:0), phytosphingosine, LysoPC (14:0), and LysoPC (18:0). Collectively, previous studies and our clinical observations imply that further studies aimed at investigating the mechanistic relationships between lipid metabolism and hyperinsulinemia as well as hyperandrogenism will be of great interest.

In order to prevent ovarian hyperstimulation syndrome, the amount of gonadotropin in the PCOS group was relatively less than the control group. However, because the number of basal antral follicles was significantly higher than that in the control group, the number of eggs obtained in the PCOS group was relatively higher. Therefore, it is necessary to further confirm that the observed changes in follicular fluid were resulted from the alterations due to PCOS or differences in gonadotropins treatment. In the further study, we will evaluate the relationship between gonadotropin dosage and changes in follicular fluid composition.

## Conclusions

Using advanced SWATH™-based mass spectrometry, we investigated metabolic changes in the follicular fluid of women with PCOS. We report the first clinical data showing that PCOS patients have distinct metabolic profiles, the components of which could be potentially applied as biomedical markers for diagnosing PCOS. Specifically, the levels of free fatty acids, 3-hydroxynonanoyl carnitine and eicosapentaenoic acid were significantly higher, whereas the levels of lysophosphatidylcholines (LysoPC) (16:0), phytosphingosine, LysoPC (14:0), and LysoPC (18:0) were significantly lower in women with PCOS. Additionally, the levels of steroid hormone deoxycorticosterone and two amino acids, phenylalanine and leucine, were higher in the PCOS patients. Our findings reveal that metabolic profiles differ between PCOS phenotypes and control subjects, and these differences may enable the development of important biochemical information and metabolic signatures that will improve understanding of the pathogenesis of PCOS.

## Data Availability

The datasets used and analyzed during the current study are available from the corresponding author on reasonable request.

## Abbreviations

DAG: Isotope-labeled 15:0–18:1(d7) diglyceride
IDA: Information dependent acquisition
LPC: Isotope-labeled d3-palmitic acid and Isotope-labeled 18:1(d7) lysophosphatidylcholine
LPE: Isotope-labeled 18:1(d7) lysophosphatidylethanolamine
LysoPC: Lysophosphatidylcholines
MAG: Isotope-labeled 18:1(d7) monoglyceride
MS: Mass spectrometry
PCA: Principal component analysis
PCOS: Polycystic ovary syndrome
PLS-DA: Partial least squares-discriminant analysis
QC: Quality control
RSD: Relative standard deviation
SM: Isotope-labeled 18:1(d9) sphingomyelin
SWATH™: Sequential window acquisition of all theoretical fragment-ion spectra
TCA: Tricarboxylic acid
UPLC-Q-TOF: Ultra-performance liquid chromatography quadrupole time-of-flight mass spectrometry
VIP: Variable importance in the project
